# The inflammatory effects of TNF-α and complement component 3 on coagulation

**DOI:** 10.1038/s41598-018-20220-8

**Published:** 2018-01-29

**Authors:** Martin J. Page, Janette Bester, Etheresia Pretorius

**Affiliations:** 1 Department of Physiological Sciences, Stellenbosch University, Stellenbosch, Private Bag X1, MATIELAND, 7602 South Africa; 2 Department of Physiology, University of Pretoria, Pretoria, 0007 South Africa

## Abstract

Tissue necrosis factor-α (TNF-α) and complement component 3 (C3) are two well-known pro-inflammatory molecules. When TNF-α is upregulated, it contributes to changes in coagulation and causes C3 induction. They both interact with receptors on platelets and erythrocytes (RBCs). Here, we look at the individual effects of C3 and TNF-α, by adding low levels of the molecules to whole blood and platelet poor plasma. We used thromboelastography, wide-field microscopy and scanning electron microscopy to study blood clot formation, as well as structural changes to RBCs and platelets. Clot formation was significantly different from the naïve sample for both the molecules. Furthermore, TNF-α exposure to whole blood resulted in platelet clumping and activation and we noted spontaneous plasma protein dense matted deposits. C3 exposure did not cause platelet aggregation, and only slight pseudopodia formation was noted. Therefore, although C3 presence has an important function to cause TNF-α release, it does not necessarily by itself cause platelet activation or RBC damage at these low concentrations. We conclude by suggesting that our laboratory results can be translated into clinical practice by incorporating C3 and TNF-α measurements into broad spectrum analysis assays, like multiplex technology, as a step closer to a patient-orientated, precision medicine approach.

## Introduction

Tissue necrosis factor-α (TNF-α) and complement component 3 (C3) are two well-known pro-inflammatory molecules^1^. They are not only upregulated in most inflammatory conditions, but their activities are closely linked. When TNF-α is upregulated, it contributes to changes in coagulation and C3 induction^2^. TNF-α plays a pivotal role in the disruption of macrovascular and microvascular circulation both *in vivo* and *in vitro*^3,4^, and is an important cytokine that can induce both apoptosis and inflammation^5^. In the presence of ROS, there is an increased production of TNF-α and, in turn, TNF-α signaling accentuates oxidative stress^3^. TNF-α upregulation is also associated with a changed coagulation propensity^6,7^. In short, TNF-α participates in vasodilatation and oedema formation, as well as leukocyte adhesion to the epithelium through expression of adhesion molecules. Furthermore, it regulates blood coagulation, contributes to oxidative stress at sites of inflammation, and indirectly induces fever^6^. TNF-α also plays a central role in the pathogenesis of insulin-resistant metabolic derangements.

C3 is a major protein of innate immunity and the complement system, with crucial roles in microbial killing, apoptotic cell clearance, immune complex handling and modulation of adaptive immune responses^8^. The relationship between upregulation of C3, inflammation, coagulation and hypofibrinolysis has been known for a long time^9,10^. Furthermore, pathology in the complement system is associated with an increased risk of pathological thrombotic processes^11^ e.g. stroke^12^ and in the pathogenesis of thromboembolism^13^.

C3 and TNF-α activity are interlinked, and examples of complement-dependent TNF release has been found in viral fulminant hepatitis^2^, atherosclerosis-induced inflammation^14^, rheumatoid arthritis^15^, human umbilical cord vein (HUVEC) endothelial cells^16^, and in insulin resistance^17^. It was also found that TNF-α synthesis by *Mycobacterium avium* is modulated through complement-dependent interaction *via* complement receptors 3 and 4 in relation to *M. avium* glycopeptidolipid^18^. TNF-α may also induce epithelial to mesenchymal transition in injured renal tubular epithelial cells through inducing C3 expression^19^. Table 1 shows concentrations of both TNF-α and C3 in health and disease cited in the literature.

**Table 1:** Circulating concentrations of TNF-α and C3 in health and disease.

Reference ranges for circulating C3 and TNF-α levels in humans		
TNF-α levels in controls	5.6 ± 2.0 pg·ml^−1^	^48^
	13.4 ± 0.81 pg·ml^−1^ in males and 13.9 ± 4.5 pg·ml^−1^ in females	^4^
	14.80 ± 4.74 pg·ml^−1^	^49^
	3.9 ± 2. 5 pg·ml^−1^	^50^
	0.7 ± 0.2 pg·ml^−1^	^51^
TNF-α levels in disease	Type 2 diabetes: 4.28 ± 5.01 pg·mL^−1^	^52^
	Chronic liver failure, TNF-α was found to be 53.50 ± 73.49 pg·mL^−1^ amongst survivors	^49^
	Metabolic syndrome: 6.3 ± 1.9 pg·ml^−1^	^48^
	Aortic stenosis patients: 2.1 ± 1.6 pg·ml^−1^and mitral regurgitation: 1.3 ± 0.7 pg·ml^−1^	^51^
C3 levels in controls	Healthy individuals 18 years and older: 88–201 mg·dL^−1^	^53^
	88.5 ± 19 mg·dL^−1^	^54^
	95 (94.5–95.5) mg·dL^−1^	^55^
C3 levels in disease	Prediabetes: 103.2 (102.5–03.8) mg·dL^−1^	^55^
	Diabetes: 1.35 (1.10–1.60) g·L^−1^	^56^

These two circulating pro-inflammatory molecules affect the coagulation and haematological system, as they interact with receptors on both platelets and erythrocytes (RBCs). Tumor necrosis factor receptors 1 and 2 (TNFR1; TNFR2) are the primary TNF-α receptors^20^, with TNFR1, also known as tumor necrosis factor receptor superfamily member 1A (TNFRSF1A) or CD120a, being the chief receptor. While RBCs do not express these receptors^21^, TNF can induce platelet consumption, and platelets do express TNFR1 and TNFR2^22,23^. TNFR1 expressed on other cells also causes the release of factors with agonist activity for platelets^24^; and TNF-α is able to activate platelets through stimulation of the arachidonic acid pathway^22,23^.

Turning to complement, RBCs carry the complement receptor 1 (CR1), also known as C3b/C4b receptor or CD35, on its membrane^25^. Immune complexes, which have reacted with complement and bear C3b fragments also bind to the CR1 on human RBCs, and CR1 on RBCs serves as a transport system for immune complexes in the circulation to prevent immune complex deposition outside the fixed macrophage system^26–28^. Complement also interacts with the surface of activated platelets as well as with other components of the complement system including, C1q, C4, C3, and C9, which bind to activated platelets^11,29^. Furthermore, thrombin-activated platelets can actually initiate the complement cascade^29,30^, and C3a and its derivative C3a-des-Arg, induce platelet activation and aggregation *in vitro*^31^. Platelets express complement receptors C3aR, CR4, as well as a receptor for iC3b and C5a, and the C1q receptors gC1qR and cC1qR on their membranes. cC1qR, in particular, was shown to mediate platelet aggregating and activating effects^29^. Of importance is that platelets may also interact with the complement system *via* proteins that are not considered classical complement receptors, such as P-selectin^32^ or GP1bα^33^.

In a series of papers, we have investigated the individual effects of inflammatory molecules on coagulation^34,35^. During inflammation, various circulating inflammatory molecules are upregulated, and a crucial result of this combination of molecules is a pathological haematological system that ultimately translates to hypercoagulation, RBC dysfunction and platelet hyperactivation – all hallmarks of inflammation and cytokine upregulation. However, for clinical intervention, it is essential to know what the individual effects on these pro-inflammatory molecules are, to pinpoint possible biochemical interventions. Here we look at the individual effects of C3 and TNF-α, by adding low levels of the molecules to blood.

## Materials and Methods

### Ethical statement

The ethics committees of the University of Pretoria and Stellenbosch University (South Africa) approved this study (ethics clearance number 298/2016). A written form of informed consent was obtained from all healthy donors (available on request). The methods were carried out in accordance with the approved guidelines. Blood was collected and methods were carried out in accordance with the relevant guidelines of the ethics committee. We adhered strictly to the Declaration of Helsinki.

### Sample and blood collection

Blood was collected from 14 healthy individuals who voluntarily enrolled for this study. Exclusion criteria for the healthy population were: known (chronic and acute) inflammatory conditions such as asthma, human immunodeficiency virus (HIV) or tuberculosis; risk factors associated with metabolic syndrome; smoking; and, if female, being on contraceptive or hormone replacement treatment. This population did not take any anti-inflammatory medication. Based on satisfying the exclusion criteria, the control donors were classified as ostensibly healthy. We therefore assumed that the TNF-α and C3 levels in our chosen sample population were in the ranges previously reported by researchers (see Table 1). Our added final concentrations of the two products therefore slightly increased their intrinsic levels to simulate a state of low-grade chronic inflammation.

Blood was collected in citrate tubes and a plasma poor isolate was derived by centrifuging whole blood for 15 minutes at 3000 *g*. Platelet poor plasma was stored at − 80 °C prior to experimentation.

### Products: TNF-α and C3

We exposed whole blood and plasma separately to either TNF-α (Sigma T6674) or C3 (Sigma C2910) at levels that represent low-grade chronic inflammation. Our final TNF-α exposure concentration in blood and plasma was 1 pg·mL^−1^ and our final C3 exposure concentration was 0.0025 mg·mL^−1^. We exposed blood to higher concentrations of TNF-α **(**15 and 30 pg·mL^−1^ final exposure concentration) and also to a higher final exposure concentration of 0.2 mg.mL^−1^ C3. These higher concentrations did not allow a clot to be formed on the TEG, as we could not obtain a clot R-time, suggesting that the added high concentrations were causing the clot to form too fast. However, we do believe that we simulated low-grade chronic inflammation with our low TNF-α and C3 final exposure concentration; but we do recognise that the physiological ranges during disease can be much higher.

### Thromboelastography of whole blood and platelet poor plasma

Viscoelastic assessment of clot kinetics was performed using thromboelastography (TEG). Whole blood (WB) and platelet poor plasma (PPP) from healthy donors was incubated with TNF-α or C3 for 10 minutes prior to assessment. WB was left at room temperature for 15 minutes following collection before being incubated. PPP was first thawed to room temperature from storage at −80 °C before incubation. 340 μL of naïve (untreated) or product-exposed WB or PPP was mixed with 20 μl of 0.2 M CaCl_2_ in a disposable TEG cup. Recalcification of blood is necessary to reverse the effect of the sodium citrate collecting tube anticoagulation method and consequently initiate coagulation. The samples were then placed in the computer-controlled Thromboelastograph® 5000 Hemostasis Analyzer System (Haemonetics Inc., Braintree, MA, USA) for analysis at 37 °C. Table 2 summarises the seven clot parameters that were studied^36–38^.

**Table 2:** TEG clot parameters for whole blood and platelet poor plasma (adapted from^38^).

Parameters	Explanation
R value: reaction time; measured in minutes	Time of latency from start of test to initial fibrin formation (amplitude of 2 mm); i.e. initiation time
K value: kinetics; measured in minutes	Time taken to achieve a certain level of clot strength (amplitude of 20 mm); i.e. amplification
Angle (Α/Alpha): slope between the traces represented by R and K; measured in degrees	The angle measures the speed at which fibrin build up and cross linking takes place, hence assesses the rate of clot formation; i.e. thrombin burst
Maximal Amplitude (MA): measured in mm	Maximum strength/stiffness of clot. Reflects the ultimate strength of the fibrin clot, i.e. overall stability of the clot
Maximum rate of thrombus generation (MRTG): measured in Dyn.cm^−2^.s^−1^	The maximum velocity of clot growth observed or maximum rate of thrombus generation using G, where G is the elastic modulus strength of the thrombus in dynes per cm^−2^
Time to maximum rate of thrombus generation (TMRTG): measured in minutes	The time interval observed before the maximum speed of the clot growth
Total thrombus generation (TTG): measured in Dyn.cm^−2^	The clot strength: the amount of total resistance (to movement of the cup and pin) generated during clot formation. This is the total area under the velocity curve during clot growth, representing the amount of clot strength generated during clot growth

### Wide-field microscopy of whole blood using the Zeiss CellDiscoverer 7

RBC autofluorescence was used to follow the effects of the products added to WB. Images were acquired with the Zeiss CellDiscoverer 7 using a combination of three LED modules simultaneously: 385, 470 and 567 nm wavelengths. The following bandpass emission filters were used: 412–438; 501–527 and 583–601. A Plan-Apochromat 50×/1.2NA objective with a 2× tube lens and an Axiocam 506 mono-camera were used for image acquisition. The live cell imaging procedure included adding 99 μL of WB in a plate and imaging the naïve sample. Then, 1 μL of either TNF-α or C3 was added, and following stabilisation of the sample, images were captured at 3 minutes after exposure.

### Scanning electron microcopy of whole blood

Blood smears were prepared for SEM analysis using WB after exposure to TNF-α and C3 for 10 minutes. In short, 10 μL of unexposed naïve and product-exposed WB were placed on glass cover slips and smears were washed in PBS, fixed in 4% formaldehyde and 1% osmium tetraoxide before being dehydrated in increasing grades of ethanol and HMDS (for detailed methods see^34,38^). Micrographs of WB smears were taken using a Zeiss crossbeam electron microscope and Merlin (Gemini II) FE SEM to study the ultrastructure of the both platelets and RBCs, with specific focus on their membranes. Image acquisition was performed independently at both the Universities of Stellenbosch and Pretoria. The images acquired at both these institutions are stored as raw data (see link below).

### Statistical analysis

TEG parameters were analysed by the repeat measures One-Way ANOVA with the Holm-Sidak test (and this includes corrections such as the Greenhouse-Geisser correction for sphericity/equal variability of differences). This type of analysis allows us to compare each product exposure with the control (GraphPad Prism 7), with statistical significance taken as p ≤ 0.05.

### Data sharing

Raw data, extensive SOPs for TEG and SEM, including original images without color and micrographs can be accessed at: https://1drv.ms/f/s!AgoCOmY3bkKHuh5Av5hYQU5UQgdG. Raw data can also be access at the corresponding author’s researchgate link: https://www.researchgate.net/profile/Etheresia_Pretorius.

## Results

### Thromboelastography of whole blood and platelet poor plasma

WB TEG parameters reflect clot properties due to both the cellular components (viz. platelets and RBC), as well as the plasma protein components^38^; while PPP clot results reflect only the effect of the plasma proteins, chiefly fibrin(ogen). Table 3 shows sample demographics and TEG results for both WB and PPP, with significant p-values in bold.

**Table 3:** Sample demographics and TEG results for both WB and PPP before and after exposure to TNF-α or C3. Values presented as median ± standard deviation.

Demographic Data of Healthy Individuals				N = 14; Age 24.0 ± 12.2; M = 57%; F = 43%	
TEG results for naïve whole blood exposed to TNF-α or C3 for 10 minutes at room temperature					
	Naïve (n = 8)	TNF-α (n = 8)	Naïve vs TNF-α P-value	Complement 3 (n = 8)	Naïve vs C3 P-value
R	9.30 ± 1.47	8.40 ± 1.09	0.077	8.85 ± 0.93	<0.0001
K	3.80 ± 0.95	3.45 ± 1.02	0.16	3.55 ± 1.04	0.24
Angle	45.10 ± 5.37	48.50 ± 7.57	0.16	47.20 ± 7.66	0.16
MA	52.25 ± 5.43	54.35 ± 8.03	0.96	53.30 ± 5.32	0.76
MRTG	3.35 ± 0.79	3.82 ± 1.03	0.17	3.89 ± 1.06	0.17
TMRTG	13.84 ± 3.49	12.54 ± 3.66	0.37	13.17 ± 2.46	0.45
TTG	547.03 ± 141.48	596.87 ± 150.22	0.86	594.38 ± 127.38	0.77
**TEG results for naïve platelet poor plasma exposed to TNF-α or C3 for 10 minutes at room temperature**					
	**Naïve (n = 11)**	**TNF-α (n = 11)**	**Naïve vs TNF-α P-value**	**Complement 3 (n = 11)**	**Naïve vs C3 P-value**
R	13.20 ± 3.31	11.80 ± 1.97	0.03	10.60 ± 2.58	0.03
K	4.50 ± 2.31	4.60 ± 1.31	0.60	4.80 ± 2.17	0.94
Angle	43.60 ± 13.73	47.80 ± 8.55	0.11	52.00 ± 10.00	0.12
MA	23.80 ± 5.51	24.60 ± 4.92	0.99	22.90 ± 6.32	0.91
MRTG	3.11 ± 1.35	3.16 ± 1.62	0.05	3.41 ± 1.75	0.03
TMRTG	13.92 ± 3.51	13.33 ± 2.33	0.02	11.83 ± 3.18	0.13
TTG	155.64 ± 56.98	164.16 ± 54.24	0.83	148.62 ± 64.15	0.83

### Wide-field microscopy using the Zeiss CellDiscoverer 7

**Figure 1:**
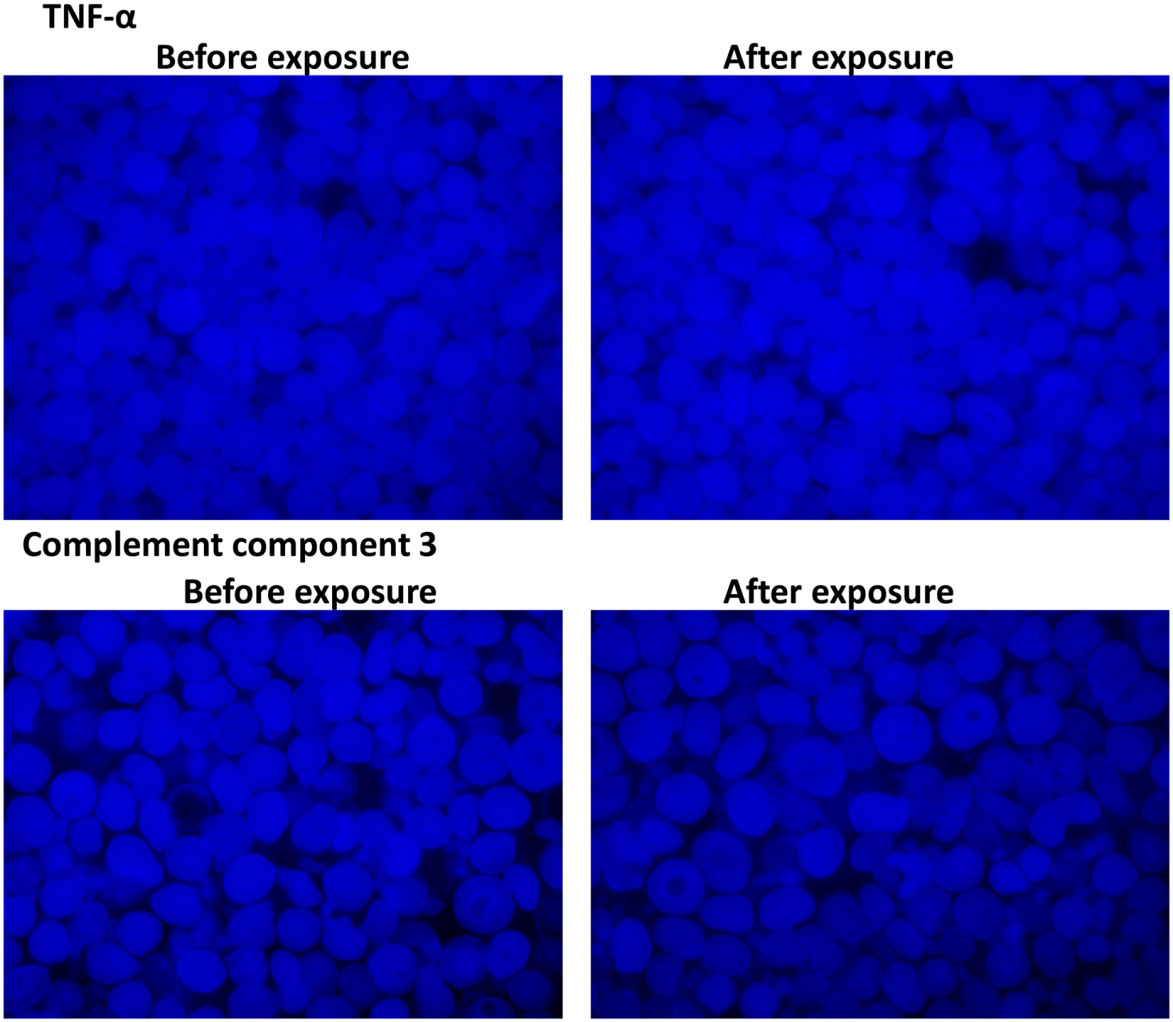
Wide-field microscopy using the Zeiss CellDiscoverer 7, before and 3 minutes after exposure to TNF-α or C3.

Figure 1 shows the wide-filed microscopy results before and after exposure to TNF-α and C3. This equipment gives us the option to, in real-time, observe changes as the inflammatory molecules are added. We could not detect any changes to RBCs after exposure to the low physiological concentrations of the products. We followed up this experiment with scanning electron microscopy, where we could look at cellular interactions between platelets and RBCs and at membrane changes in these cells, using high magnification.

### Scanning electron microscopy of whole blood

**Figure 2:**
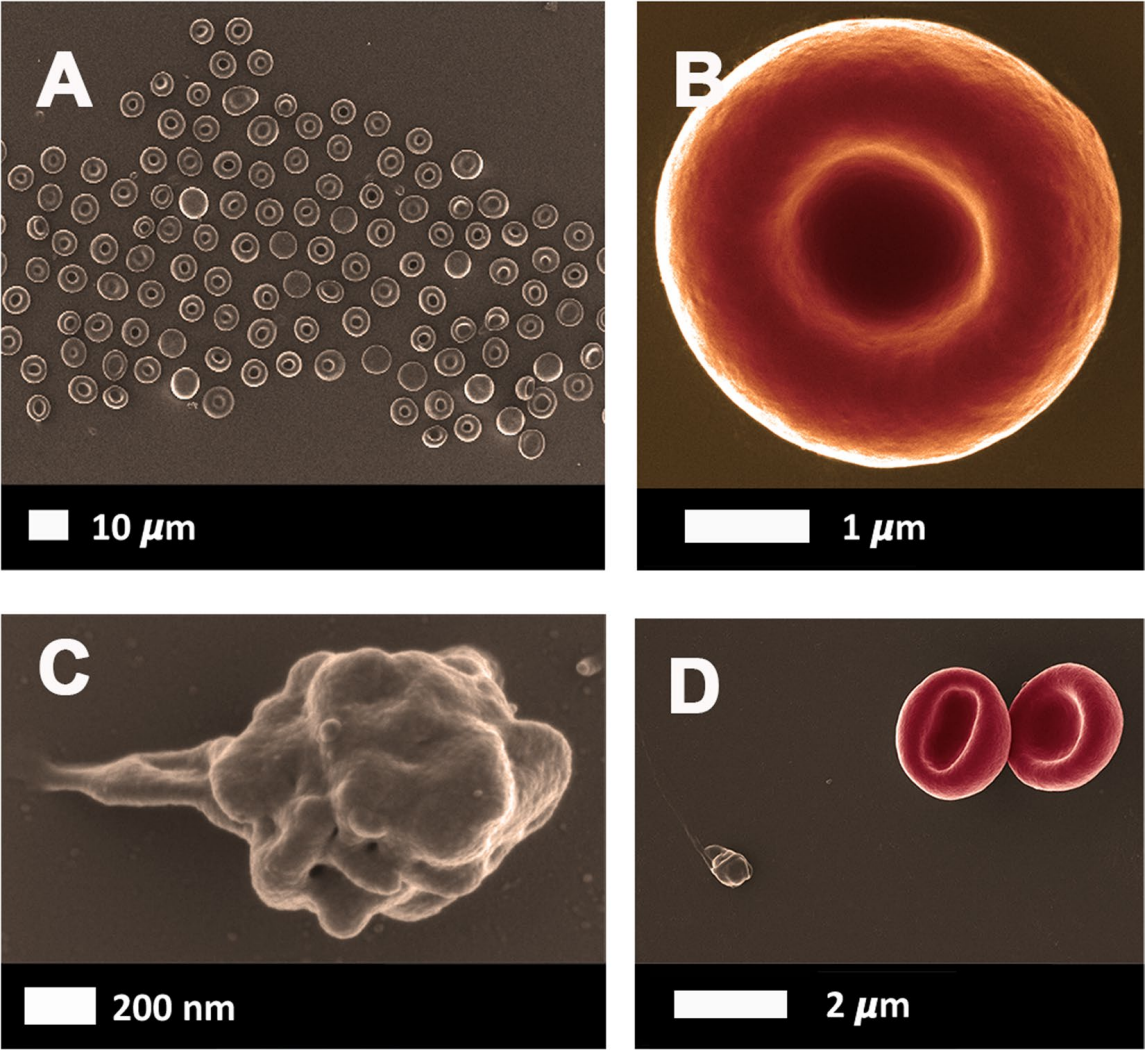
(**A**) Low magnification of a healthy whole blood smear prepared for scanning electron microscopy. (**B**) A representative RBC and (**C**) a representative platelet. (**D**) RBCs and platelet showing little activation.

**Figure 3:**
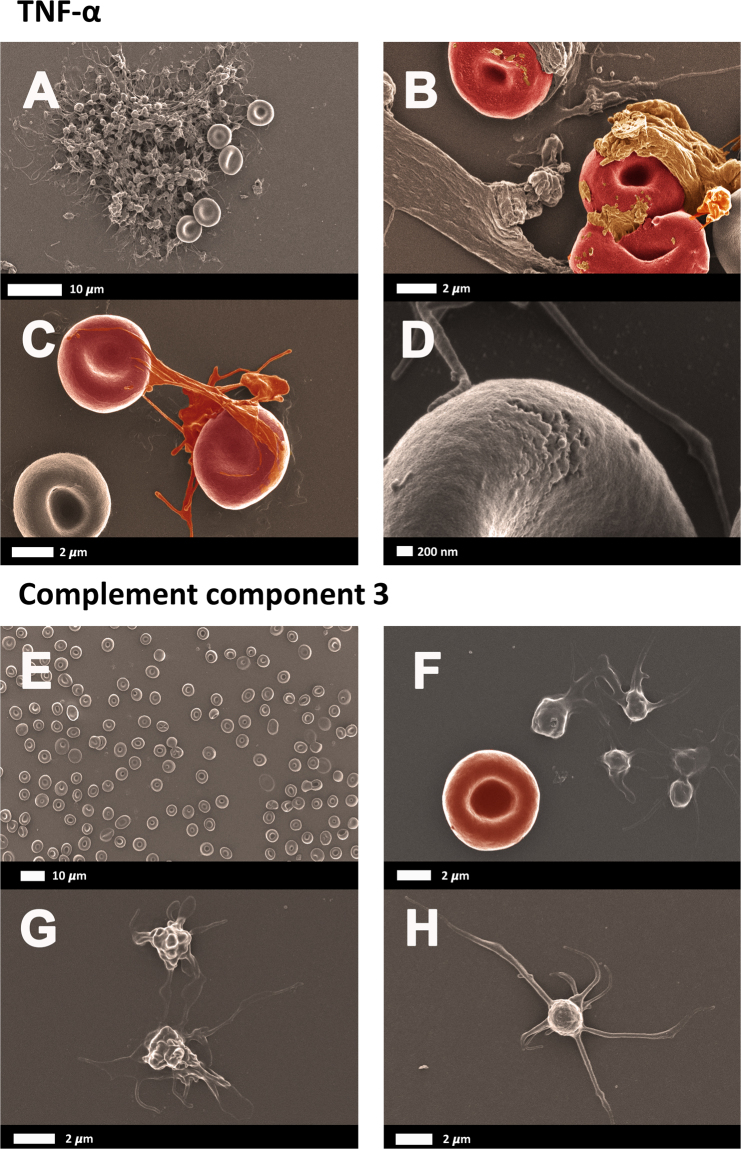
(**A** to **D)** Healthy whole blood smear after exposure to TNF-α and then prepared for scanning electron microscopy. (**E** to **H)** Healthy whole blood exposed to complement component 3.

Figure 2A shows a typical healthy whole blood smear, at low magnification, and Fig. 2B and C, higher magnification of RBCs and platelets. Figure 3 shows micrographs from healthy whole blood exposed to TNF-α and C3. TNF-α exposure resulted in both RBC damage and platelet hyeractivation and clumping, while C3 exposure only resulted in a slightly increased platelet pseudopodia formation.

## Discussion

During inflammation, various circulating inflammatory molecules are upregulated, and a crucial result of this combination of molecules is a pathological haematological system that ultimately translates to hypercoagulation, RBC dysfunction and platelet hyperactivation – all hallmarks of inflammation and cytokine upregulation. However, for clinical intervention, it is essential to know what the individual effects on these pro-inflammatory molecules are, to pinpoint possible biochemical interventions. Here we look at the individual effects of C3 and TNF-α, by adding low levels of the molecules to blood. In a series of papers, we have investigated the individual effects of inflammatory molecules on coagulation^34,35^.

In our previous research we noted that the presence of all inflammatory molecules such as IL-1ß, IL-6, Il 8 and Il-12 cause platelets to be hyperactivated, clumped or aggregated^34,35,39^. In the current investigation, only TNF-α exposure, resulted in platelet clumping and activation (See Fig. 3A). TNF-α exposure also resulted in spontaneous plasma protein dense matted deposits, that surrounded and covered RBCs and showed interactions with platelet pseudopodia (Fig. 3B and C). C3, at the physiological levels that we used, did not cause platelet aggregation, and only slight pseudopodia formation was noted. This is an important observation, as C3 and TNF-α activity are interlinked, and examples of complement-dependent TNF-α release are well-known, as discussed in the introduction. Therefore, although C3 presence has an important function to cause TNF-α release, it does not necessarily by itself cause platelet activation.

Increased levels of TNF-α are known to activate platelets^40,41^, and during inflammation, complement is activated on the surface of platelets, despite the presence of multiple regulators of complement activation^11^. A recent paper also showed that C3 plays specific roles in platelet activation^42^. The authors showed, in a tissue factor-dependent model of flow restriction-induced venous thrombosis, that complement factors play a role in platelet activation and fibrin deposition^42^. In our paper, we did not additionally quantitatively assess platelet activation in the presence of our chosen molecules, but showed the effect visually using SEM.

We have also previously shown that RBCs may be affected by cytokines and that they can become eryptotic in the presence of cytokines like IL-8^34^ or agglutinated in the presence of IL-12^39^. In the current investigation, we did not observe any eryptosis, however, TNF-α did cause membrane changes and this was visible as membrane disintegration (see Fig. 3D). This was, however, not present in the majority of the cells. C3 did not cause any structural membrane changes after exposure. The results were confirmed with the wide-field microscopy results.

SEM allows the visualization of samples at a very detailed resolution. With TNF-α exposure, the RBC morphology did not appear to be the primary change. Rather, the plasma proteins appeared be changed and covered some of the RBCs. Also, with SEM we noted greater platelet reactivity. Since the wide-field visualization was based only on RBC auto-florescence and that it is not as sensitive as SEM, these changes were also not readily apparent with this technique.

Our TEG results showed that there were no significant differences in naïve whole blood when compared with whole blood samples with added TNF-α; although the R-time showed a trend towards a faster clotting initiation time (p = 0.077). When C3 was added to whole blood, the R-time shorted significantly. Overall these two products seem to promote a faster clot initiation. There were significant differences in the TEG results when platelet poor plasma (PPP) was analysed. It is known that the diagnosis of hypercoagulability are based on changes in variables of hemostatic monitors such as a decrease in the time to clot initiation (reaction time, R), an increase in the speed of clot propagation (angle), or an increase in clot strength (amplitude, A; or shear elastic modulus G). This indicates an enhancement of hemostasis or hypercoagulability as defined with TEG^43,44^. Here we show a significant decrease in the R-time, MRTG and the TMRTG with PPP and added TNF-α. Also a significant decrease in R-time and a significant increase in MRTG with PPP and added C3. According to the results, the PPP samples with the added C3 and TNF-α showed that more parameters point towards hypercoagulation, thus suggesting these molecules have a more profound effect on fibrin formation.

Defining and understanding the role of cytokines and other inflammatory mediators, like TNF-α and C3 in (inflammatory) diseases, is becoming increasingly important, especially in patient-orientated and precision-based therapy initiatives^45^. We conclude by suggesting that our laboratory results can be translated into clinical practice by incorporating C3 and TNF-α measurements into broad spectrum analysis assays, like multiplex technology, that measures various cytokines (e.g. interleukins). Developments in inflammatory marker quantification technology, like multiplex assays and arrays, allow for an improved evaluation and understanding of the dynamic nature of inflammatory responses^46,47^. Furthermore, multiplexed protein array assays provide high sensitivity and specificity using low sample volumes in a high throughput analysis and, in addition, can be used during therapeutic drug monitoring. With such approaches, therapy outcomes can also be followed with more accuracy, and in the long run, reduce treatment costs, as well as expedite wellness outcomes.

Considering that our results show that these two molecules have a pro-coagulant effect on blood, in addition to a place in diagnostics, it might be of importance to evaluate further the biochemical mechanisms by which C3 and TNF-α modulate pro-coagulant pathways. This might reveal a therapeutic potential for lowering the pro-coagulant state due to C3 and TNF-α, present in inflammatory diseases.
